# Comparative evaluation of certain biomarkers emphasizing abnormal GABA inhibitory effect and glutamate excitotoxicity in autism spectrum disorders

**DOI:** 10.3389/fpsyt.2025.1562631

**Published:** 2025-04-22

**Authors:** Altaf Alabdali, Abir Ben Bacha, Mona Alonazi, Laila Y. Al-Ayadhi, Abdullah S. J. Alanazi, Afaf El‐Ansary

**Affiliations:** ^1^ Biochemistry Department, Science College, King Saud University, Riyadh, Saudi Arabia; ^2^ Autism Research and Treatment Center, Department of Physiology, Faculty of Medicine, King Saud University, Riyadh, Saudi Arabia; ^3^ Biochemistry Laboratory, Qurayyat General Hospital, Qurayyat, Saudi Arabia; ^4^ Autism Center, Lotus Holistic Alternative Medical Center, Abu Dhabi, United Arab Emirates

**Keywords:** autism spectrum disorder (ASD), gamma-aminobutyric acid (GABA), glutamate, KCC2, NKCC1, EAAT2, vitamin D3 (VD3), GABRA5

## Abstract

**Introduction:**

Autism spectrum disorder (ASD) is a neurodevelopmental disorder characterized by social communication deficits and repetitive behaviors. An imbalance between the excitatory neurotransmitter glutamate and the inhibitory neurotransmitter gamma-aminobutyric acid (GABA) might play a crucial role in ASD. This study explores the biochemical markers associated with GABAergic and glutamatergic signaling in individuals with autism and healthy controls, aiming to identify potential diagnostic and therapeutic targets.

**Methods:**

The study included 46 male individuals with autism and 26 age- and gender-matched healthy controls. The plasma levels of excitatory amino acid transporter 2 (EAAT2), potassium chloride co-transporter 2 (KCC2), Na–K–Cl co-transporter 1 (NKCC1), vitamin D3 (VD3), GABA, gamma aminobutyric acid type a receptor subunit alpha 5 (GABRA5), and glutamate were measured using ELISA. Statistical analyses, including correlation, multiple regression, and receiver operating characteristic (ROC) curve analysis, were performed to evaluate the diagnostic utility and interrelationships of these biomarkers.

**Results:**

Significant biochemical differences were found between individuals with autism and healthy controls. Individuals with autism had notably lower levels of EAAT2, KCC2, NKCC1, VD3, GABA, and GABRA5, especially in the severe group. Altered KCC2/NKCC1 and GABA/glutamate ratios highlighted the imbalance in neurotransmission. The correlation and multiple regression analyses showed significant interconnections between biomarkers. The ROC analysis indicated that EAAT2, KCC2, GABA, and the ratios of KCC2/NKCC1 and GABA/glutamate have high diagnostic potential.

**Conclusion:**

These findings support the hypothesis that GABA and glutamate imbalance is central to the pathophysiology of ASD. Significant disruptions in neurotransmitter signaling and chloride homeostasis, particularly in severe cases, provide insights into the neurobiological mechanisms of ASD. Restoring the GABA–glutamate balance could be an effective therapeutic strategy for ASD, warranting further research into these biochemical pathways for targeted treatments.

## 1 Introduction

Autism spectrum disorder (ASD) refers to a series of neurodevelopmental disorders characterized by social communication deficits and repetitive behaviors (1). Individuals with autism often have other conditions, such as intellectual disability, mood disorders, epilepsy, and gastrointestinal issues (2–4). The prevalence of autism has risen over time and is more common in male than in female individuals, with an estimated 1 in 100 children worldwide affected (5, 6). In Riyadh, Saudi Arabia, the prevalence is 2.51%, with a male-to-female ratio of 3:1 (7). Numerous studies have shown that genetic, epigenetic, and perinatal environmental factors, as well as their complex interactions, are involved in the neurophysiological mechanisms of autism (8–10). As the etiology of this disorder remains unknown, neither a specific treatment nor reliable diagnostic biomarkers are currently available. Due to the rapidly increasing frequency of ASD, there is an urgent need to discover diagnostic biomarkers (11).

Neurotransmitters are crucial for the development of both the central and peripheral nervous systems, and their dysfunction may be implicated in ASD. Gamma-aminobutyric acid (GABA) and glutamate homeostasis are vital for brain function and are affected in various neuropsychiatric disorders (12–14). Evidence suggests that an imbalance between excitatory (glutamate) and inhibitory (GABA) neurotransmission may be a key pathophysiological mechanism and a potential treatment target in ASD (15–18).

The excitatory/inhibitory (E/I) imbalance hypothesis, proposed by Rubinstein and Merzenich (15), suggests that autism symptoms result from an imbalance between the excitatory (glutamatergic) and inhibitory (GABAergic) brain mechanisms. Studies have identified causes such as overexcitation and overinhibition (15, 19–21). Understanding how E/I imbalance underlies autism symptoms is complex; however, evidence suggests that genetic and environmental risk factors disrupt the balance of excitatory and inhibitory neurotransmission. This disruption could help identify therapeutic targets for autism (19, 22, 23).

GABA is the primary inhibitory neurotransmitter in the brain, and its receptors play a crucial role in modulating neuronal excitability (23–25). Dysfunction in GABA receptors has been implicated in autism (15, 26–28). Studies have shown that individuals with autism often exhibit alterations in GABAergic signaling, which can contribute to the imbalance between excitation and inhibition in the brain (29–33). For instance, reduced activity of the GABA type A (GABA_A_) receptors has been observed in the brain of individuals with autism, which may lead to increased neuronal excitability and contribute to sensory hypersensitivity and other symptoms associated with autism (34, 35). During and after brain development, neuronal Cl^−^ control plays a role in the dynamic regulation of GABAergic inhibition. This regulation depends mainly on two cation chloride co-transporters, such as sodium potassium chloride co-transporter 1 (NKCC1) and potassium chloride co-transporter 2 (KCC2), to regulate the intracellular chloride concentration, which in turn affects GABAergic signaling (36, 37). NKCC1 accumulates chloride inside neurons, making GABA an excitatory neurotransmitter during early development. In contrast, KCC2 reduces the intracellular chloride, making GABA inhibitory in mature neurons (38). Abnormalities in the expression or function of these transporters can disrupt GABAergic signaling (39). Studies have indicated that the developmental switch from NKCC1 to KCC2 might be delayed or dysfunctional in individuals with autism, resulting in improper inhibitory signaling and contributing to autism-related symptoms (40–43).

Glutamate is the primary excitatory neurotransmitter in the brain, and its transporters, such as excitatory amino acid transporter 1 (EAAT1) and transporter 2 (EAAT2), are responsible for the maintenance of the extracellular glutamate levels (44–46). Dysregulation of glutamate transporters can lead to excitotoxicity and neuronal damage (47–49). Research has shown that individuals with autism often have an altered glutamate transporter activity, which leads to increased extracellular glutamate levels and excitotoxicity. This E/I imbalance is thought to contribute to the neurological and behavioral symptoms observed in autism (22, 50).

Vitamin D plays a crucial role in the development and function of the brain (51). It regulates the expression of several genes involved in neurotransmission, neuroprotection, and neurodevelopment (52). There is growing evidence suggesting that vitamin D deficiency may be linked to the development and severity of autism (53–57). Some studies have indicated that adequate levels of vitamin D could help modulate GABAergic and glutamatergic signaling, potentially alleviating some of the symptoms of autism (58–60).

The purpose of this study was to assess factors related to glutamate excitotoxicity in plasma samples from individuals with autism in comparison to healthy controls. These factors include imbalanced GABA/glutamate levels resulting from dysfunction in the GABA receptors, chloride co-transporters, and glutamate transporters together with vitamin D deficiency. Through this study, it may be possible to ascertain how these biomarkers might influence the severity and the clinical presentation of ASD and how to best target them for treatment.

## 2 Materials and methods

### 2.1 Participants

The participants in this study were recruited from the Autism Research and Treatment Centre at King Khalid University Hospital Riyadh, King Saud University, Kingdom of Saudi Arabia. A total of 46 autistic male patients and 26 age- and gender-matched controls were recruited. All participants were screened and evaluated using the Diagnostic and Statistical Manual of Mental Disorders IV (DSM-IV). Scores were calculated to subclassify the participants into mild, moderate, or severe using the Childhood Autism Rating Scale (CARS) and the Social Responsiveness Scale (SRS).

### 2.2 Behavioral assessment

#### 2.2.1 Childhood Autism Rating Scale

The CARS is a widely utilized screening tool for autism (61). It consists of 15 observational domains designed to distinguish ASD from other developmental conditions in children. The scale rates each domain on a continuum from 1 (indicative of typical behavior) to 4 (indicative of severe abnormality), with higher scores reflecting greater levels of impairment. The domains assessed include interpersonal relations, emotional responses, imitation abilities, use of body and objects, listening skills, fear or anxiety, verbal and nonverbal communication, activity level, intellectual response, adaptability to change, sensory responses (such as visual, taste, smell, and touch), and overall impressions. Total scores can range from 15 to 60, with scores below 30 suggesting a non-autistic range, scores between 30 and 36.5 indicating mild-to-moderate autism, and scores between 37 and 60 reflecting severe autism.

#### 2.2.2 Social Responsiveness Scale

The SRS is a 65-item tool used to quantify the severity of autistic traits (62). It is a structured questionnaire completed by parents or teachers, which takes approximately 15–20 min, based on the child’s behavior observed over the previous 6 months. The SRS employs a standard four-point scale, where responses range from “0” (not true) to “3” (almost always true). The assessment is divided into five subscales: social awareness, social cognition, social communication, social motivation, and autistic mannerisms. An SRS score between 60 and 75 suggests mild-to-moderate social impairment, while a score of 76 or above indicates severe social difficulties.

### 2.3 Ethical approval

Institutional Review Board and Guidelines of Health Sciences Colleges Research on Human Subjects, King Saud University, College of Medicine, approved this study (no. 22/0122/IRB). An informed consent form was obtained from the parents or legal guardians of all participants prior to participation for approval for processing and publishing data. All experiments were performed in accordance with the relevant guidelines and regulations.

### 2.4 Blood samples

After an overnight fast, 10 ml blood samples were collected from both groups in test tubes containing sodium heparin as an anticoagulant. The tubes were centrifuged at 3,500 rpm at room temperature for 15 min and the resulting plasma frozen (at −80(C) until further analysis.

### 2.5 Biochemical analysis

#### 2.5.1 Determination of excitatory amino acid transporter 2

The human EAAT2 ELISA kit (ELK 4324, Wuhan, China) was used in a quantitative sandwich enzyme immunoassay. The microtiter plate was pre-coated with an EAAT2-specific antibody. After addition of the standards or samples and a biotin-conjugated EAAT2 antibody, avidin–horseradish peroxidase (HRP) was added and incubated. The reaction was stopped with sulfuric acid, and the color change was measured at 450 nm. The detection range for EAAT2 was 0.32–20 ng/ml.

#### 2.5.2 Determination of potassium chloride co-transporter 2

The levels of KCC2 were measured using an ELISA kit (ELK Biotechnology, ELK 0484, Wuhan, China). The assay used a sandwich format with a microtiter plate pre-coated with a monoclonal KCC2 antibody. The standards or samples were added to form antigen–antibody complexes. After washing, a biotin-conjugated KCC2 antibody and streptavidin–HRP were added. A substrate solution then produced a colorimetric reaction, with a detection range of 0.32–20 ng/ml.

#### 2.5.3 Determination of sodium potassium chloride co-transporter 1

The human NKCC1 ELISA kit (ELK 0483, Wuhan, China) was used to quantitatively determine the NKCC1 concentrations in plasma using a sandwich enzyme immunoassay. The microtiter plate was pre-coated with an NKCC1-specific antibody. After addition of the standards or samples, a biotin-conjugated NKCC1 antibody and avidin–HRP were added. The reaction was stopped with sulfuric acid, and the color change was measured at 450 nm. The detection range was 0.32–20 ng/ml.

#### 2.5.4 Determination of vitamin D3

The human VD3 ELISA kit (ELK 0811, Wuhan, China) was used in a competitive inhibition enzyme immunoassay. The microtiter plate was pre-coated with vitamin D3 (VD3) protein. After addition of the standards or samples and a biotin-conjugated VD3 antibody, avidin–HRP was added and incubated. The reaction was stopped with sulfuric acid, and the color change was measured at 450 nm. The detection range was 6.25–400 ng/ml.

#### 2.5.5 Determination of gamma-aminobutyric acid

The human GABA ELISA kit (ELK 0753, Wuhan, China) was used in a competitive inhibition enzyme immunoassay. The microtiter plate was pre-coated with GABA protein. After addition of the standards or samples and a biotin-conjugated GABA antibody, avidin–HRP was added and incubated. The reaction was stopped with sulfuric acid, and the color change was measured at 450 nm. The detection range was 31.25–2,000 pg/ml.

#### 2.5.6 Determination of glutamate

The plasma glutamate levels were measured using a double-sandwich ELISA kit (MyBioSource Ltd., San Diego, CA, USA). The pre-coated plate had a glutamate monoclonal antibody, and the detecting antibody was biotin-labeled. After addition of the samples and biotin-labeled antibodies, avidin–peroxidase conjugates were added. The reaction was measured at 450 nm, with a detection range of 0.312–20 nmol/ml.

#### 2.5.7 Determination of gamma-aminobutyric acid receptor subunit alpha-5

The plasma levels of gamma aminobutyric acid type a receptor subunit alpha 5 (GABRA5) were measured using a quantitative sandwich ELISA kit (MyBioSource Ltd., San Diego, CA, USA). The microtiter plate was pre-coated with a GABRA5-specific antibody. The reaction was stopped with sulfuric acid and the color change measured at 450 nm. The detection range was 0.25–8 ng/ml.

### 2.6 Statistical analysis

Statistical analysis was conducted using SPSS software (SPSS Inc., Chicago, IL, USA). Data were expressed as the mean ± SD. One-way ANOVA and the Kruskal–Wallis test were used to assess group differences, followed by the least significant difference LSD) or the Mann–Whitney test for multiple comparisons. Receiver operating characteristic (ROC) curve analysis evaluated the diagnostic performance of the biochemical parameters, reporting the area under the curve (AUC), the sensitivity, and the specificity. Parameters with high AUC values were considered strong biomarker candidates. Stepwise multiple regression was used to identify significant predictors, and standardized regression coefficients were used to assess their importance. Spearman’s correlation analysis explored the relationships between the biochemical parameters, reporting the correlation coefficients (*R*) and *p*-values.

## 3 Results

**Figure 1:**
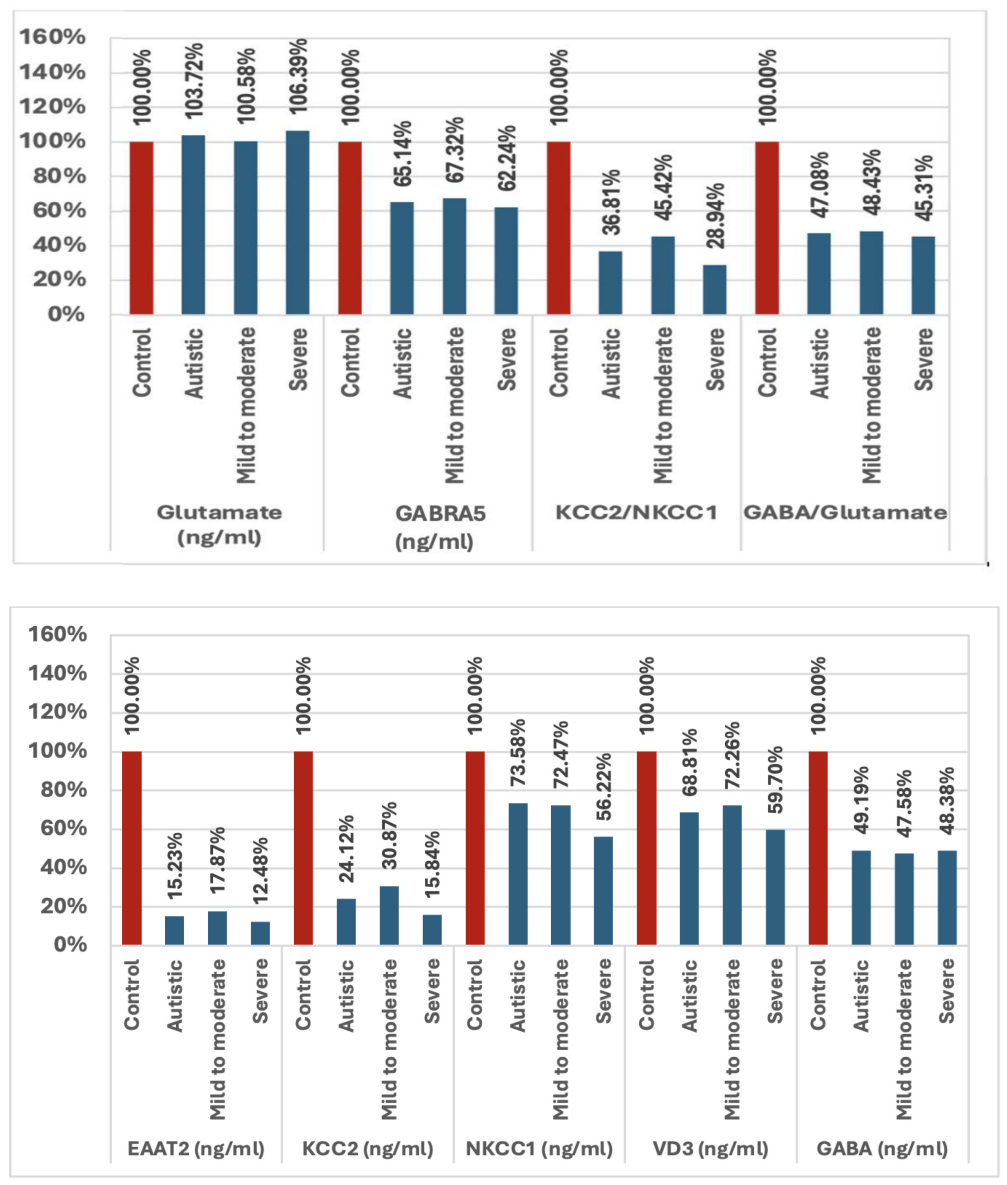
Percentage change in all variables examined in mild-to-moderate and severe participants with autism *versus* healthy controls. *EAAT2*, excitatory amino acid transporter 2; *KCC2*, potassium chloride co-transporter 2; *NKCC1*, sodium potassium chloride co-transporter 1; *VD3*, vitamin D3; *GABA*, gamma aminobutyric acid; *GABRA5*, gamma aminobutyric acid type A receptor subunit alpha 5.

**Table 1** and **Figure 1** show the primary data presented as the mean ± SD and the percent change for the measured variables. The presented data collectively highlighted significant differences between individuals with ASD and healthy controls, as well as among subgroups of ASD (i.e., mild-to-moderate and severe). The parameters EAAT2, KCC2, NKCC1, VD3, GABA, and GABRA5 exhibited markedly lower plasma levels in individuals with autism compared with controls, with the most substantial reductions observed in the severe group. The ratios of KCC2/NKCC1 and GABA/glutamate were also significantly altered in the autistic groups. While the glutamate levels were slightly elevated in individuals with autism, the increase was less pronounced than the changes observed in the other parameters.

**Table 1:** Parameters assessed in the plasma of participants with autism in comparison to healthy controls.

Parameter	Group	*N*	Min.	Max.	Mean ± SD	Median	*p*-value
EAAT2 (ng/ml)	Control	26	1.26	31.30	10.64 ± 7.55^a–c^	9.08	0.001
	Autistic	46	0.40	5.98	1.62 ± 1.10^d^	1.43	
	Mild-to-moderate	26	0.64	5.98	1.90 ± 1.31^d^	1.62	
	Severe	18	0.40	2.09	1.33 ± 0.58^d^	1.34	
KCC2 (ng/ml)	Control	26	1.08	11.90	4.92 ± 3.27^a–c^	4.18	0.001
	Autistic	46	0.51	5.55	1.19 ± 1.01^d^	0.74	
	Mild-to-moderate	26	0.54	5.55	1.52 ± 1.21^c,d^	1.21	
	Severe	18	0.51	1.81	0.78 ± 0.37^b,d^	0.68	
NKCC1 (ng/ml)	Control	26	2.12	28.50	10.96 ± 6.72^a–c^	10.04	0.035
	Autistic	46	2.06	47.40	8.07 ± 7.08^d^	6.93	
	Mild-to-moderate	26	2.36	17.70	7.95 ± 4.35^d^	7.99	
	Severe	18	2.06	12.30	6.16 ± 2.87^d^	6.38	
VD3 (ng/ml)	Control	26	3.54	30.90	15.89 ± 8.01^a–c^	15.53	0.041
	Autistic	46	0.00	30.90	10.93 ± 8.87^d^	10.78	
	Mild-to-moderate	26	0.00	30.90	11.48 ± 8.61^d^	10.78	
	Severe	18	0.00	30.90	9.49 ± 9.26^d^	9.59	
GABA (ng/ml)	Control	26	0.023	0.204	0.124 ± 0.052^a–c^	0.119	0.001
	Autistic	46	0.003	0.147	0.061 ± 0.036^d^	0.0611	
	Mild-to-moderate	26	0.0123	0.140	0.059 ± 0.034^d^	0.054	
	Severe	18	0.003	0.147	0.060 ± 0.040^d^	0.064	
Glutamate (ng/ml)	Control	26	13,600	20,500	17,104.00 ± 1,699.50	17,061	0.530
	Autistic	46	11,900	25,200	17,741.00 ± 2,961.46	17,580	
	Mild-to-moderate	26	11,900	25,200	17,204.00 ± 3,261.16	16,780	
	Severe	18	14,000	25,200	18,197.00 ± 2,943.45	17,580	
GABRA5 (ng/ml)	Control	26	1.22	15.10	4.18 ± 3.03^a–c^	3.35	0.003
	Autistic	46	0.64	6.01	2.72 ± 1.04^d^	2.72	
	Mild-to-moderate	26	0.64	6.01	2.81 ± 1.24^d^	2.72	
	Severe	18	0.95	4.18	2.60 ± 0.96^d^	2.72	
KCC2/NKCC1	Control	26	0.214	1.760	0.525 ± 0.365^a–c^	0.435	0.001
	Autistic	46	0.043	0.851	0.193 ± 0.172^d^	0.144	
	Mild-to-moderate	26	0.057	0.851	0.239 ± 0.208^d^	0.168	
	Severe	18	0.043	0.395	0.152 ± 0.088^d^	0.119	
GABA/glutamate	Control	26	1.58E−06	1.22E−05	7.37E−06 ± 3.30E−06^a–c^	6.85E−06	0.001
	Autistic	46	1.97E−07	8.56E−06	3.47E−06 ± 2.05E−06^d^	3.47E−06	
	Mild-to-moderate	26	8.08E−07	8.56E−06	3.57E−06 ± 2.18E−06^d^	3.51E−06	
	Severe	18	1.97E−07	7.23E−06	3.34E−06 ± 2.09E−06^d^	3.49E−06	

**Table 1** displays the one-way ANOVA results between different groups with multiple comparisons (LSD test) within the entire groups for each parameter (for parametric data) and the Kruskal–Wallis test between different groups with multiple comparisons (Mann–Whitney test) within the entire groups for each parameter (for non-parametric data).

EAAT2, excitatory amino acid transporter 2; KCC2, potassium chloride co-transporter 2; NKCC1, sodium potassium chloride co-transporter 1; VD3, vitamin D3; GABA, gamma aminobutyric acid; GABRA5, gamma aminobutyric acid type a receptor subunit alpha 5.

**^a^** Describes significant difference between the group and the (autistic group) at the significant level (0.05).

**^b^** Describes significant difference between the group and the (mild-to-moderate group) at the significant level (0.05).

**^c^** Describes significant difference between the group and the (severe group) at the significant level (0.05).

**^d^** Describes significant difference between the group and the (control group) at the significant level (0.05).

**Table 2** presents the correlations between the different biochemical parameters using Spearman’s correlation, showing correlation coefficients (*R*) and *p*-values. EAAT2 showed strong positive correlations with KCC2, NKCC1, GABA, KCC2/NKCC1, and GABA/glutamate. KCC2 was also correlated positively with NKCC1, GABA, GABRA5, KCC2/NKCC1, and GABA/glutamate. NKCC1 showed negative correlations with glutamate, but a positive correlation with GABRA5. VD3 was positively correlated with GABA, GABRA5, KCC2/NKCC1, and GABA/glutamate. GABA had a very strong positive correlation with GABA/glutamate.

**Table 2:** Spearman’s correlations among all the measured variables.

Parameter	*R* (correlation coefficient)	*p*-value	
EAAT2 ~ KCC2	0.743**	0.001	P^a^
EAAT2 ~ NKCC1	0.341**	0.001	P^a^
EAAT2 ~ GABA	0.212*	0.022	P^a^
EAAT2 ~ KCC2/NKCC1	0.482**	0.001	P^a^
EAAT2 ~ GABA/glutamate	0.247**	0.008	P^a^
KCC2 ~ NKCC1	0.402**	0.001	P^a^
KCC2 ~ GABA	0.249**	0.007	P^a^
KCC2 ~ GABRA5	0.279**	0.002	P^a^
KCC2 ~ KCC2/NKCC1	0.667**	0.001	P^a^
KCC2 ~ GABA/glutamate	0.288**	0.002	P^a^
NKCC1 ~ glutamate	−0.223*	0.016	N^b^
NKCC1 ~ GABRA5	0.276**	0.003	P^a^
NKCC1 ~ KCC2/NKCC1	−0.312**	0.001	N^b^
VD3 ~ GABA	0.312**	0.001	P^a^
VD3 ~ glutamate	0.172	0.065	P^a^
VD3 ~ GABRA5	0.248**	0.007	P^a^
VD3 ~ KCC2/NKCC1	0.286**	0.002	P^a^
VD3 ~ GABA/glutamate	0.265**	0.004	P^a^
GABA ~ GABRA5	0.245**	0.008	P^a^
GABA ~ KCC2/NKCC1	0.398**	0.001	P^a^
GABA ~ GABA/glutamate	0.968**	0.001	P^a^
Glutamate ~ GABA/glutamate	−0.102	0.057	N^b^
GABRA5 ~ GABA/glutamate	0.197*	0.034	P^a^
KCC2/NKCC1 ~ GABA/glutamate	0.395**	0.001	P^a^

EAAT2, excitatory amino acid transporter 2; KCC2, potassium chloride co-transporter 2; NKCC1, sodium potassium chloride co-transporter 1; VD3, vitamin D3; GABA, gamma aminobutyric acid; GABRA5, gamma aminobutyric acid type A receptor subunit alpha 5.

*p = 0.05 level; **p = 0.01.

a P, Positive correlation.

b N, Negative correlation.

The results of the multiple regression analyses highlighted significant predictors for various dependent variables, demonstrating the complex biochemical interactions in ASD. For EAAT2 as the dependent variable (**Table 3**), KCC2 was identified as a significant predictor, explaining 49.6% of the EAAT2 variance with a coefficient of 1.578. In contrast, KCC2 as the dependent variable (**Table 4**) was influenced by EAAT2, KCC2/NKCC1, NKCC1, and GABRA5 as independent variables, collectively accounting for up to 79% of its variance. NKCC1 (**Table 5**) was strongly predicted by KCC2 and the KCC2/NKCC1 ratio, indicating their crucial roles in the regulation of NKCC1. The VD3 levels (**Table 6**) were significantly associated with GABA, albeit explaining a modest 8.8% of its variance. The GABA/glutamate ratio (**Table 7**) emerged as a primary positive predictor for the GABA levels, accounting for 98.3% of its variance, while GABRA5 also played a notable role. Similarly, KCC2 (**Table 8**) was a significant predictor of GABRA5, explaining 6.5% of its variance. The KCC2/NKCC1 ratio (**Table 9**) was positively influenced by KCC2 and negatively by NKCC1, explaining up to 68.7% of its variance. Finally, the GABA/glutamate ratio and GABRA5 (**Table 10**) were significant predictors for GABA/glutamate, with the former explaining up to 98.3% of the variance and the latter showing a smaller but a significant negative effect.

**Table 3:** Multiple regression using the stepwise method for EAAT2 as a dependent variable.

Predictor variable	Coefficient	SE	*p*-value	Adjusted *R* ^2^	95% CI	
					Lower	Upper
KCC2	1.578	0.148	0.001	0.496	1.285	1.870

EAAT2, excitatory amino acid transporter 2; KCC2, potassium chloride co-transporter 2; CI, confidence interval.

**Table 4:** Multiple regression using the stepwise method for KCC2 as a dependent variable.

Predictor variable	Coefficient	SE	*p*-value	Adjusted *R* ^2^	95% CI	
					Lower	Upper
EAAT2	0.317	0.030	0.001	0.496	0.258	0.376
EAAT2	0.217	0.029	0.001	0.635	0.159	0.276
KCC2/NKCC1	3.866	0.580	0.001		2.717	5.014
EAAT2	0.127	0.025	0.001	0.782	0.077	0.176
KCC2/NKCC1	5.350	0.479	0.001		4.400	6.300
NKCC1	0.164	0.019	0.001		0.127	0.201
EAAT2	0.124	0.024	0.001	0.790	0.076	0.173
KCC2/NKCC1	5.255	0.472	0.001		4.320	6.189
NKCC1	0.158	0.019	0.001		0.121	0.194
GABRA5	0.134	0.057	0.020		0.021	0.246

EAAT2, excitatory amino acid transporter 2; KCC2, potassium chloride co-transporter 2; NKCC1, sodium potassium chloride co-transporter 1; GABRA5, gamma aminobutyric acid type A receptor subunit alpha 5; CI, confidence interval.

**Table 5:** Multiple regression using the stepwise method for NKCC1 as a dependent variable.

Predictor variable	Coefficient	SE	*p*-value	Adjusted *R* ^2^	95% CI	
					Lower	Upper
KCC2	1.066	0.221	0.001	0.162	0.628	1.504
KCC2	2.504	0.231	0.001	0.512	2.046	2.962
KCC2/NKCC1	−18.635	2.048	0.001		−22.693	−14.577
KCC2	2.462	0.227	0.001	0.532	2.012	2.912
KCC2/NKCC1	−18.423	2.007	0.001		−22.400	−14.446

KCC2, potassium chloride co-transporter 2; NKCC1, sodium potassium chloride co-transporter 1; CI, confidence interval.

**Table 6:** Multiple regression using the stepwise method for VD3 as a dependent variable.

Predictor variable	Coefficient	SE	*p*-value	Adjusted *R* ^2^	95% CI	
					Lower	Upper
GABA	0.056	0.016	0.001	0.088	0.024	0.089

VD3, vitamin D3; GABA, gamma aminobutyric acid; CI, confidence interval.

**Table 7:** Multiple regression using the stepwise method for GABA as a dependent variable.

Predictor variable	Coefficient	SE	*p*-value	Adjusted *R* ^2^	95% CI	
					Lower	Upper
GABA/glutamate	16,799.372	215.483	0.001	0.983	16,372.42	17,226.32
GABRA5	0.831	0.341	0.016		0.156	1.506

GABA, gamma aminobutyric acid; GABRA5, gamma aminobutyric acid type A receptor subunit alpha 5; CI, confidence interval.

**Table 8:** Multiple regression using the stepwise method for GABRA5 as a dependent variable.

Predictor variable	Coefficient	SE	*p*-value	Adjusted *R* ^2^	95% CI	
					Lower	Upper
KCC2	0.208	0.069	0.003	0.065	0.071	0.345

*GABRA5*, gamma aminobutyric acid type A receptor subunit alpha 5; *KCC2*, potassium chloride co-transporter 2; *CI*, confidence interval.

**Table 9:** Multiple regression using the stepwise method for KCC2/NKCC1 as a dependent variable.

Predictor variable	Coefficient	SE	*p*-value	Adjusted *R* ^2^	95% CI	
					Lower	Upper
KCC2	0.077	0.008	0.001	0.463	0.062	0.092
KCC2	0.101	0.006	0.001	0.687	0.089	0.114
NKCC1	−0.023	0.002	0.001		−0.028	−0.018

KCC2, potassium chloride co-transporter 2; NKCC1, sodium potassium chloride co-transporter 1; CI, confidence interval.

**Table 10:** Multiple regression using the stepwise method for GABA/glutamate as a dependent variable.

Predictor variable	Coefficient	SE	*p*-value	Adjusted *R* ^2^	95% CI	
					Lower	Upper
GABA/glutamate	5.85E−05	7.50E−07	0.001	0.983	5.70E−05	5.99E−05
GABRA5	−4.24E−05	2.02E−05	0.039		−8.24E−05	−2.28E−06

GABA, gamma aminobutyric acid; GABRA5, gamma aminobutyric acid type a receptor subunit alpha 5; CI, confidence interval.

The ROC analysis evaluated the diagnostic accuracy of specific biomarkers in distinguishing individuals with autism from controls and among different autism severity levels. For the autistic group compared with the controls, **Table 11** shows that EAAT2 (AUC = 0.952) had a sensitivity of 93.5% and a specificity of 84.6% (*p* = 0.001), while KCC2 (AUC = 0.931) had a sensitivity of 89.1% and a specificity of 88.5% (*p* = 0.001), indicating their strong diagnostic potential. The KCC2/NKCC1 ratio (AUC = 0.883) had a sensitivity of 73.9% and a specificity of 100.0% (*p* = 0.001), while the GABA/glutamate ratio (AUC = 0.836) showed a sensitivity of 84.8% and a specificity of 69.2% (*p* = 0.001). Another notable biomarker was GABA (AUC = 0.827, sensitivity = 84.8%, specificity = 73.1%, *p* = 0.001).

**Table 11:** ROC results for the autistic group according to the control group.

Parameter	AUC	Cutoff value	Sensitivity (%)	Specificity (%)	*p*-value	95% CI
EAAT2	0.952	3.331	93.5	84.6	0.001	0.903–1.000
KCC2	0.931	1.860	89.1	88.5	0.001	0.876–0.987
NKCC1	0.664	9.553	80.4	57.7	0.019	0.527–0.801
VD3	0.671	13.326	67.4	65.4	0.008	0.545–0.796
GABA	0.827	0.092	84.8	73.1	0.001	0.727–0.927
Glutamate	0.539	15,707.00	21.7	96.2	0.564	0.327–0.594
GABRA5	0.646	3.058	78.3	61.5	0.053	0.498–0.793
KCC2/NKCC1	0.883	0.210	73.9	100.0	0.001	0.807–0.959
GABA/glutamate	0.836	0.006	84.8	69.2	0.001	0.738–0.934

ROC, receiver operating characteristic; AUC, area under the curve; CI, confidence interval; EAAT2, excitatory amino acid transporter 2; KCC2, potassium chloride co-transporter 2; NKCC1, sodium potassium chloride co-transporter 1; VD3, vitamin D3; GABA, gamma aminobutyric acid; GABRA5, gamma aminobutyric acid type A receptor subunit alpha 5.

## 4 Discussion

This study showed significant biochemical differences between individuals with autism and healthy controls, with specific emphasis on the subgroups of autism (mild-to-moderate and severe). These findings help to enhance our understanding of the neurobiological mechanisms of autism, aligning with previous research while offering new insights. Individuals with autism, particularly those in the severe group, exhibited reduced levels of EAAT2, KCC2, NKCC1, VD3, GABA, and GABRA5. These disruptions in neurotransmitter signaling and chloride homeostasis likely contribute to the E/I imbalance observed in ASD. The modulation of glutamate homeostasis is most recently being investigated and has been highlighted as a potential critical target for the treatment of neurodevelopmental disorders, among which is ASD (63).

This study showed significantly lower levels of EAAT2 in individuals with autism, especially in the severe group. Although the glutamate levels were slightly elevated, the change was less pronounced. This aligns with research indicating that EAAT2 dysregulation leads to extracellular glutamate accumulation and excitotoxicity. Rothstein et al. (64) found that beta-lactam antibiotics upregulated EAAT2, which reduced the toxicity of glutamate, suggesting their potential therapeutic benefits for ASD. Moreover, EAAT2 modulation, as a therapeutic approach, alleviated the symptoms of glutamate dysregulation in mouse models of neurodegeneration (65). These findings underscore the crucial function of EAAT2 in autism and suggest that the regulation of its expression could aid in managing the neurobiological problems in ASD by resolving the E/I imbalance.

This study found significantly lower levels of KCC2 and NKCC1 in individuals with autism, particularly in the severe subgroup. KCC2 maintains low intracellular chloride for GABAergic inhibition, while NKCC1 accumulates chloride, making GABA excitatory. The dysregulation of these transporters likely contributes to the E/I imbalance in ASD. Duarte et al. (66) showed that altered KCC2 and NKCC1 expression affected neuronal Cl^−^ gradients, impacting GABAergic inhibition. Adjustment of these transporters can reverse GABA polarity, exacerbating the symptoms of ASD. Clinical trials using the NKCC1 inhibitor bumetanide improved autistic behaviors, supporting the therapeutic potential of targeting these transporters (67). These findings suggest that the modulation of KCC2 and NKCC1 could offer a new therapeutic approach for ASD.

The levels of VD3 were significantly lower in individuals with autism, supporting previous research linking vitamin D deficiency to an increased risk of ASD (68, 69). This study highlighted the role of VD3 in the neurodevelopment and neurotransmitter systems, suggesting that supplementation could benefit the management of autism. Guo et al. (53) found that VD3 deficiency worsened the neuroinflammatory responses in autism. Two studies demonstrated the neuroprotective effects of VD3, showing behavioral improvements with supplementation (59, 70). El-Ansary (22) reported that vitamin D deficiency decreased the glutamic acid decarboxylase levels, disrupting the glutamate–glutamine–GABA cycle associated with autism, potentially reducing glutamate excitotoxicity. These findings suggest a promising approach for therapeutic interventions targeting VD3 deficiency in ASD.

This study found significant reductions in GABA and its receptor GABRA5 in individuals with autism, especially in the severe subgroup, indicating that disruptions in GABAergic signaling are critical for maintaining neuronal balance. This aligns with previous studies reporting decreased GABAergic activity in autism (29, 71). Fatemi et al. (34) found similar reductions in GABAergic markers, highlighting consistent GABAergic dysfunction in autism. Lower GABRA5 levels suggest a compromised neurotransmission, contributing to the E/I imbalance in ASD. Previous studies (72, 73) showed that the GABA_A_ receptor subtypes, particularly the α5 subunit, are associated with autistic behaviors, supporting our findings on disrupted GABA signaling.

The ratios of KCC2/NKCC1 and GABA/glutamate were significantly altered in the autistic groups, especially in the severe subgroup, supporting the E/I imbalance hypothesis by Rubenstein and Merzenich (15). Several studies further support these findings, linking abnormalities in the GABA and glutamate levels to the sensory and behavioral issues in ASD (19, 32, 74). Altered KCC2/NKCC1 ratios indicate a disrupted chloride homeostasis, impairing GABAergic neurotransmission and contributing to ASD symptoms. Duarte et al. (66) suggest that targeting this imbalance could offer new therapeutic perspectives.

The results of the correlation analysis (**Table 2**) revealed significant interconnections between the biochemical parameters in individuals with autism. A strong positive correlation between EAAT2 and the KCC2/NKCC1 ratio suggests efficient glutamate clearance as EAAT2 is linked to neuronal E/I balance, mediated by KCC2 and NKCC1. This supports the findings by Duarte et al. (66) indicating that alterations in the glutamate transporter activity are linked to shifts in the E/I balance in the brain. The positive correlation between KCC2 and the GABA/glutamate ratio indicates that higher levels of KCC2 enhance GABAergic inhibition, aligning with the research by Sernagor et al. (75) suggesting that boosting the function of KCC2 can correct the E/I imbalance seen in neurodevelopmental disorders. GABA and GABRA5 also showed a positive correlation, emphasizing the regulatory mechanism between neurotransmitters and receptors, supported by Robertson et al. (32). This relationship is pivotal in stabilizing neural circuits and is often disrupted in ASD, which leads to behavioral and cognitive impairments. The significant correlations between VD3 and both GABA and GABRA5 suggest the role of VD3 in modulating GABAergic signaling, as proposed by Cannell (68). These correlations highlight potential therapeutic targets, suggesting that enhancing the function of EAAT2 or KCC2 and optimizing the levels of VD3 could mitigate the core ASD symptoms.

The ROC analysis results highlighted the diagnostic potential of the biochemical markers for ASD. EAAT2 and KCC2 showed excellent diagnostic accuracy. Green et al. (76) emphasized the role of EAAT2 in modulating synaptic glutamate and preventing excitotoxicity, which are relevant to ASD. Moreover, KCC2 is important in the maintenance of GABAergic inhibition, suggesting its potential as a therapeutic target (67). NKCC1 and VD3 had moderate AUC values, indicating their lower diagnostic reliability, but still supported as markers for ASD (36, 77). These findings suggest that EAAT2 and KCC2 are effective diagnostic markers for ASD and that targeting these markers could help treat the underlying neurochemical imbalances.

The results of the multiple regression analyses highlighted the significant relationships between these markers. KCC2 strongly predicted the EAAT2 levels, suggesting its crucial role in modulating EAAT2 expression. Two studies support the importance of KCC2 in chloride transport and synaptic inhibition, underscoring its significance in neurological disorders (78, 79). Similarly, the GABA/glutamate ratio and GABRA5 significantly predicted the GABA levels, corroborating the findings by previous studies that highlighted the complex regulatory interactions in ASD (15, 50, 73).

In the current study, it is worth noting that severe individuals had considerably more observed alterations in the variables that were measured than mild-to-moderate participants with autism (**Table 1**, **Figure 1**), showing that an altered GABA inhibition is a significant contributing factor to the severity of ASD. Several studies have revealed that hypo-inhibition and/or hyper-excitation, which boosts the E/I ratio, are the primary reasons for the social processing deficiencies, sensory sensitivity, and elevated anxiety reactivity in social contexts in autism, which are important characteristics of the disorder (80, 81). GABAergic inhibition is essential for social interactions as it filters information, regulates inhibition, and processes rewards. Moreover, pharmacologically, different parts of the brain and circuits that govern repetitive behaviors as another clinical presentation of ASD react differently to local and systemic glutamatergic inhibitors and GABAergic medications (82).

## 5 Conclusions

This study highlights the importance of biochemical parameters such as EAAT2, KCC2, NKCC1, VD3, GABA, and GABRA5 in understanding ASD. Significant disruptions in neurotransmitter signaling and chloride homeostasis, especially in severe ASD, provide insights into its neurobiological mechanisms. These findings support previous research and reveal novel aspects for future studies and therapies. The results of the multiple regression analyses underscore the potential of these parameters as therapeutic targets and biomarkers for better ASD diagnosis and intervention. Further research is needed to explore these biochemical pathways for more effective ASD treatments.

### 5.1 Limitations and future research

The small sample size, together with the absence of female participants, could be considered as selection bias and a limitation of the current study. Future research could expand the sample size and include more diverse people to confirm the generalizability of the findings. We will endeavor to implement the reviewers’ helpful recommendation of converting data from one format to another and of incorporating autism severity scores into our future investigations. Conducting longitudinal research to determine how these markers change with age or therapy regimens in people with autism is highly recommended. Further research will be important to shed light on the implications of age- and sex-related GABAergic alterations in ASD phenotypic conditions in order to design improved and more effective personalized treatments that enhance the inhibitory effects of GABA.

## Data Availability

The original contributions presented in the study are included in the article/supplementary material. Further inquiries can be directed to the corresponding authors.
